# The effects of cognitive behavioral therapy on health-related quality of life, anxiety, depression, illness perception, and in atrial fibrillation patients: a six-month longitudinal study

**DOI:** 10.1186/s40359-023-01457-z

**Published:** 2023-12-07

**Authors:** Zheng Minjie, Xie Zhijuan, Shi Xinxin, Bai Xinzhu, Qu Shan

**Affiliations:** Department of Medical Psychology, Peking University People’s Hospital, Beijing, China

**Keywords:** CBT, Quality of life, Atrial fibrillation, Anxiety, Depression, Illness perception

## Abstract

**Background:**

Atrial fibrillation (AF) often leads to an impaired Health-Related Quality of Life (HRQoL) in many patients. Moreover, psychological factors such as depression, anxiety, and illness perception have been found to significantly correlate with HRQoL. This study aims to evaluate the long-term effectiveness of Cognitive Behavioral Therapy (CBT) in enhancing HRQoL and mitigating psychological distress among AF patients.

**Methods:**

Employing a prospective, open design with pseudo-randomization, this study encompassed pre-tests, post-treatment evaluations, and a 6-month follow-up. A total of 102 consecutive patients diagnosed with paroxysmal AF were initially enrolled. Out of these, 90 were assigned to two groups; one to receive a 10-week CBT treatment specifically focusing on anxiety, and the other to receive standard care. Outcome measures were evaluated using tools such as the Item Short Form Health Survey (SF-12), General Anxiety Disorder-7 (GAD-7), Patient Health Questionnaire-9 (PHQ-9), University of Toronto Atrial Fibrillation Severity Scale (AFSS), and Brief Illness Perception Questionnaire (BIPQ). These assessments were conducted at pre-treatment, post-treatment, and at the 6-month follow-up mark. We explored the effectiveness of CBT using Generalized Estimating Equations (GEE).

**Results:**

Our analysis revealed a notable improvement in the CBT group relative to the control group. All metrics displayed consistent improvement across a 6-month duration. At the 6-month checkpoint, the CBT group exhibited a more favorable SF-12 Mental Component Score (MCS) (50.261 ± 0.758 vs. 45.208 ± 0.887, *p* < 0.001), reduced GAD-7 (4.150 ± 0.347 vs. 8.022 ± 0.423, *p* < 0.001), BIPQ (34.700 ± 0.432 vs. 38.026 ± 0.318, *p* < 0.001), and AFSS (9.890 ± 0.217 vs. 10.928 ± 0.218, *p* = 0.001) scores when compared to the TAU group. Conversely, the SF-12 PCS (44.212 ± 0.816 vs. 47.489 ± 0.960, *p* = 0.139) and PHQ-9 scores (8.419 ± 0.713 vs. 10.409 ± 0.741, *p* = 0.794) manifested no significant difference between the two groups.

**Conclusion:**

The findings suggest that CBT is effective in improving HRQoL and reducing psychological distress among patients with AF at 6 month follow-up. This highlights the potential benefits of integrating CBT into the therapeutic regimen for AF patients.

**Trial Registration:**

Retrospectively registered with ClinicalTrials.gov (NCT05716828). The date of registration : 5 June 2023.

## Introduction

Atrial fibrillation (AF), the most prevalent arrhythmia in clinical practice, impacts 3.7–4.2% of patients between the ages of 65 and 85 [1, 2]. This prevalent arrhythmia is linked to a diminished health-related quality of life (HRQoL) when compared to other cardiovascular diseases (CVDs) [3]. An impaired quality of life is a common characteristic among AF patients, however, the connection between AF symptoms and quality of life remains debatable [4]. Current guidelines suggest that antiarrhythmic drug therapy or catheter ablation can mitigate AF symptoms, but these treatments fall short in restoring HRQoL to normal levels [5, 6]. The present research on the quality of life of AF patients primarily centers around the comparison of the aforementioned treatment options, and there is no singular therapy that is universally effective for all AF patients. Hence, it would be worthwhile to investigate alternative treatments and their potential to enhance the quality of life for individuals with AF.

**Fig. 1:**
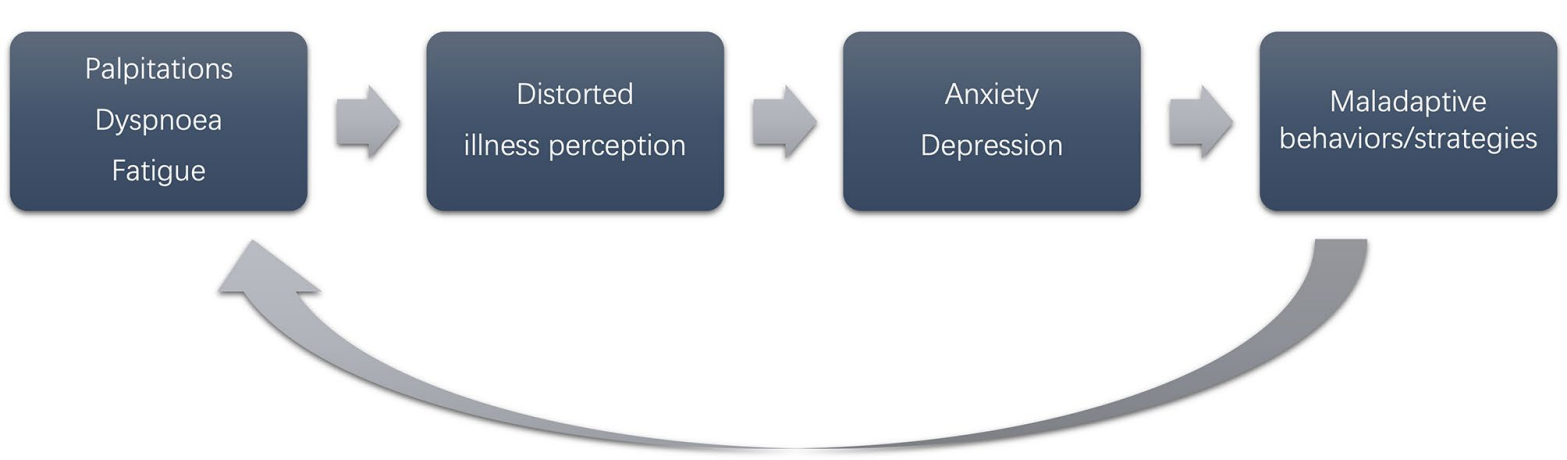
Theoretical framework for the study based on the common sense model and Beck’s CBT model

Atrial fibrillation correlates with heightened risks of anxiety and depression [7, 8]. Independent risk factors of AF recurrence after catheter ablation include anxiety [9]. Due to the anxiety and hypervigilance of AF patients towards heart-related symptoms, it is possible for them to mistake normal or stress-related heart activity as arrhythmias. Furthermore, their preoccupation with AF symptoms can potentially trigger anxiety, leading to heightened autonomic arousal, increased heart rate, and additional heartbeats that could potentially precipitate episodes of atrial fibrillation [10]. Paroxysmal atrial fibrillation (PAF) may restrict patient mobility from fear of an AF episode than due to the arrhythmia itself [11]. Factors such as symptom-related anxiety, frequency, and severity significantly influence HRQoL [3]. Furthermore, a high level of illness perception could induce maladaptive coping mechanisms and impact treatment adherence [12, 13], suggesting psychological factors can affect both clinical outcomes and HRQoL in AF patients (Fig. 1).

Cognitive-behavioral therapy (CBT) targets the cycle linking avoidance behavior, fear of symptoms, and adverse outcomes by adjusting cognition. A prior study demonstrated CBT’s positive impact on HRQoL and disease perception in AF patients suffering from depression [14]. Despite these findings, a scarcity of longitudinal studies assessing the effectiveness of CBT in AF patients. The aim of this study is to examine the acute and post-acute efficacy and feasibility of CBT, with a focus on anxiety symptoms, in patients with PAF.

## Method

### Study design

We conducted a prospective, open, pseudo-randomized study with a pretest-posttest design with a 6-month follow-up between March 2020 and December 2021 at the Department of Cardiology and Psychiatry, a comprehensive tertiary hospital in China. Potential participants were referred by a cardiologist, or presented spontaneously following our program flyers and post. We pseudo-randomized 102 enrolled patients with paroxysmal AF; in the first half of the enrollment period, all patients were assigned to the CBT group (n = 51), whereas patients recruited in the second half comprised the Treatment as Usual (TAU) group (n = 51).We excluded 12 patients who did not meet the inclusion criteria, leaving 90 patients for assignment to either group (n = 45 each). Each participant provided written informed consent. This study, retrospectively registered with ClinicalTrials.gov (NCT05716828), obtained approval from the regional ethics review board of Peking University (2020PHB151), adhering to the TREND Statement checklist for nonrandomized interventions [15].

### Participants and sample size

We set the participation criteria as: (a) age 18–75 years; (b) AF diagnosis as per the 2020 ESC Guidelines for Atrial Fibrillation, confirmed by a 12-lead ECG and cardiologist-led examination; (c) PAF diagnosis ascertained by a cardiologist, signified by a spontaneous return to normal sinus rhythm within a week [16]; and (d) ability to read and write in Chinese. Exclusion criteria included: severe complications such as unstable coronary artery disease, severe systolic dysfunction with heart failure (ejection fraction ≤ 35%), recent thoracic surgery, terminal illnesses or malignant diseases with a 1-year survival rate, psychiatric conditions inhibiting participation, regular psychological therapy for mental health conditions, participation in another study, and cognitive impairment impeding study involvement.

We determined a sample size of 60 based on detecting a significant difference in the primary outcome measure, HRQoL, between control and intervention groups [17]. Anticipating a 10% dropout rate, the sample size was adjusted to 67, with 34 participants in each group.

### Intervention and control setting

The intervention group underwent Cognitive Behavioral Therapy (CBT) focused on anxiety symptoms in addition to their usual treatment. This included ten one-hour sessions over a 10-week period, with weekly homework assignments guided by a therapist. Drawing from Beck’s CBT model of depression [18, 19], our protocol targeted two contributing factors to lower HRQoL in PAF patients: (1) excessive vigilance and fear of AF symptoms and (2) avoidance of physical and social activities due to fear. The CBT module was adjusted based on these two factors, and its contents are listed in Table 1.

**Table 1:** CBT Content (60 min/session)

Session	Aim	Procedure
1	Health education and Focus on Distorted cognition	Health education about paroxysmal atrial fibrillation, explaining the theory of mind-body interaction to patients and the principles of CBT
2	Self-awareness training	① Found ‘hot thought’, identifying automatic thinking ② Be aware of current cardiac symptoms, thoughts, feelings, and behavioral choices
3	Exposure training	Patients are invited to expose their bodies to feelings similar to atrial fibrillation symptoms (Heart rate becomes faster, etc.) by engaging in physical activities such as squatting. The purpose is to reduce patients’ fear of symptoms.
4	Habitual reversal training	Patients are encouraged to delay or turn past habitual heart rate measurement into irrelevant behaviors such as clenching fists.
5	Correcting cognitive distortion	① Disturbed ideas about AF, depression, accompanying physical ailments, and mortality were all common cognitive distortions. Education and normalization, Socratic inquiry, and A-B-C approaches used in cognitive intervention (event-thought, secondary physiological and behavioral responses) ② Evidence testing to explore the supporting and non-supporting bases for adverse cognition.
6	Behavioral activation	Allowing patients to conduct activities that are both pleasurable and can be managed for behavioral activation using the behavioral activity record table.
7	For anxiety symptoms	Education and normalization, cognitive correction, relaxation training, role playing, and a problem-solving list for cognitive reconstruction and behavioral intervention
8	Practice and feedback	Therapists acknowledge distress, normalize feelings, check mood level, activate support system, and ask about current issues utilizing reflective listening when a patient is disturbed.
9	Relapse prevention	Identifying relapse precursors, establishing a list of relapse precursors, and problem-solving training designed to increase relapse prevention and positive coping methods.
10	Review	Identifying new questions, and preparing for end of study

**Fig. 2:**
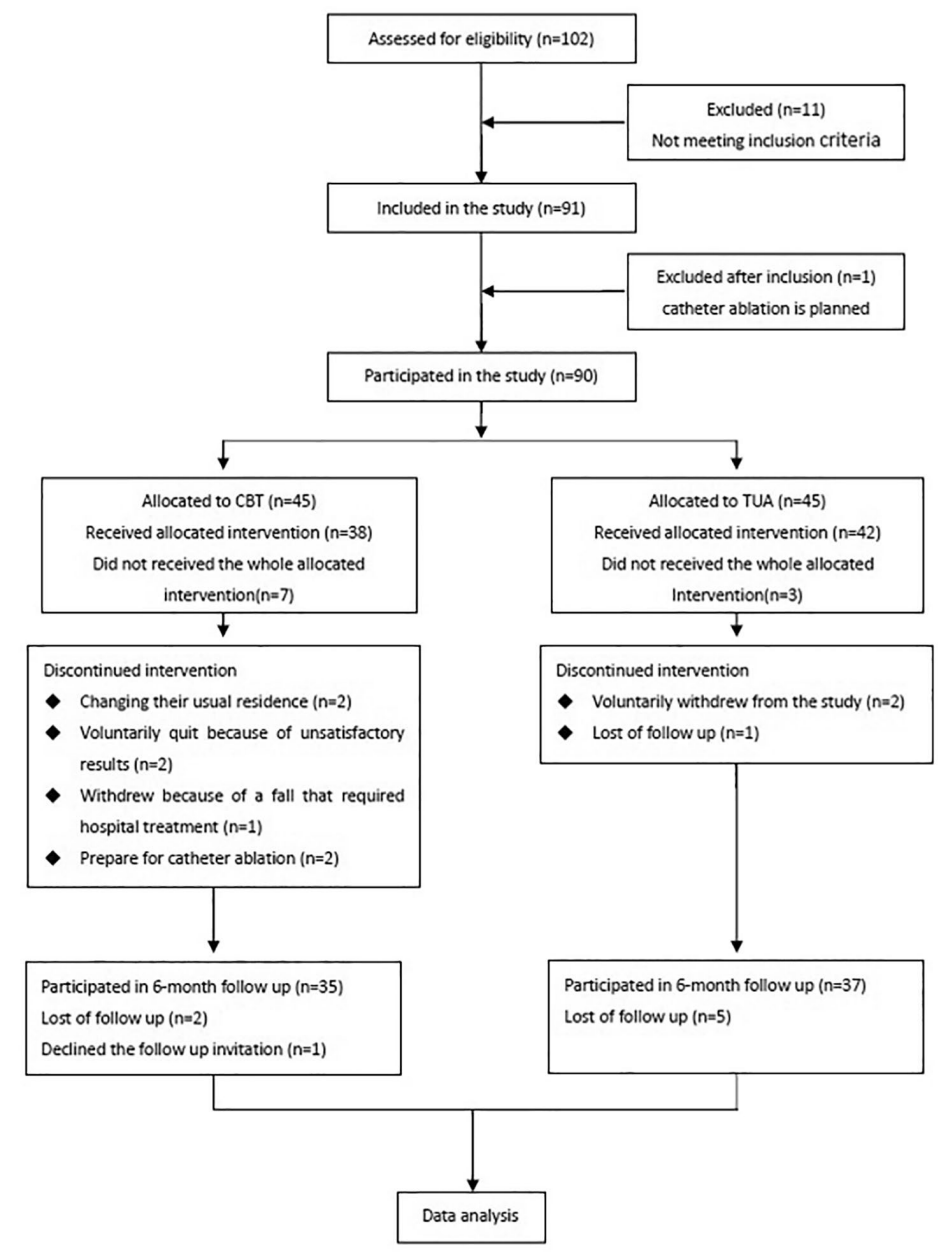
Participant flowchart

Therapy was delivered face-to-face by two trained psychologists with at least two years of CBT clinical experience. Techniques employed included goal–setting, cognitive restructuring, behavioral activation, and exposure treatment. Participants were given a handbook detailing the CBT outline, forms for recognizing automatic thoughts, and weekly assignments that encouraged patients to record their physical sensations, emotional states, thoughts, and behaviors associated with AF-related worry. Our research consort is depicted in Fig. 2.

The control group, TAU, received optimal medical care in accordance with current clinical guidelines [20]. If AF episodes heightened in frequency or provoked symptoms such as dyspnea or chest pain exacerbation, TAU patients were advised to seek immediate medical attention. They did not participate in any psychotherapy.

### Outcomes and measurements

The primary outcome was the change of HRQoL between two groups at 6 the month follow-up, as, measured by the 12-item Short Form Health Survey (SF-12). Secondary outcomes included emotional symptom changes, AF symptom alterations, and shifts in illness perception. We used the Patient Health Questionnaire-9 (PHQ-9) for assessing depressive symptoms, the Generalized Anxiety Disorder Questionnaire (GAD-7) for anxiety symptoms, the University of Toronto Atrial Fibrillation (AFSS) Symptom Subscale for AF symptoms, and the Brief Illness Perception Questionnaire (BIPQ) for evaluating illness perception.

Health-related quality of life (HRQoL) was measured using the 12-item Short Form Health Survey (SF-12), derived from the SF-36. The SF-12 incorporates two domains: physical component summary (PCS) and mental component summary (MCS), each scored from 0 to 100—higher scores denoting better health status [21].

To assess the severity of depression over the prior two weeks, we employed the nine-item Patient Health Questionnaire (PHQ-9), ranging from 0 to 27 [22]. We selected PHQ-9 due to its straightforward administration and superior performance over structured clinical interviews from the Diagnostic and Statistical Manual of Mental Disorders IV [23]. An overall score ≥ 10 indicated clinically relevant depression. The internal consistency of the PHQ-9 Chinese version was 0.82, and the test-retest reliability over a 2-week interval was 0.76 [24].

The Generalized Anxiety Disorder Questionnaire (GAD-7), a seven-item tool, measured anxiety symptoms using a four-point Likert-type scale. The internal consistency of the GAD-7 was excellent (Cronbach α = 0.92). The test-retest reliability was also good (intraclass correlation = 0.83) [25]. The GAD-7 assesses how often the patient has suffered the seven core symptoms of GAD in the last 2 weeks, with the possible responses being “Not at all”, “On some days”, “On more than half of the days” and “Almost every day” (scored 0–3, respectively, with a total score ranging from 0 to 21). An overall score ≥ 10 indicated clinically relevant anxiety.

We used the Brief Illness Perception Questionnaire (BIPQ), a nine-item tool, to gauge patients’ perceptions of their illness across nine categories. A composite BIPQ score—higher scores indicating a larger psychological burden of illness—was calculated from the individual domain scores (range:0–80). The BIPQ assesses the following illness perception domains: identity (symptoms experienced); timeline-acute/chronic (perception of length of disease); consequences (effect of disease on one’s life); personal control (control over disease); treatment control (perception of treatment impact); emotional representations (emotional effect of disease); illness coherence (understanding of disease); illness concern (concern about disease); and cause (perceived cause of disease). The cause item is an open-ended question which asks patients to rank the top three factors they believe caused their disease. The overall score was calculated by adding the score of each item, with reversed scores applied to items 3, 4, and 7. The total possible score was between 0 and 80, with higher scores indicating more negative feelings about the disease [26]. The Chinese version of the questionnaire had good psychometric characteristics, with Cronbach α = 0.77 [27].

The University of Toronto Atrial Fibrillation Severity Scale (AFSS), a disease-specific scale, comprises three parts. We chose the symptom component from Part 3 to assess the AF symptoms, including seven common items (heart palpitations, dyspnea at rest, dyspnea with activity, exercise intolerance, vertigo, fatigue at rest, and chest pain). In this questionnaire, the severity of each symptom is rated on a 6-point scale, ranging from 0 to 5 points. The total score thus can range from 0 to 35 points, with higher scores indicating more severe AF symptoms.This scale demonstrates internal consistency with a Chronbach’s α of 0.67 for health care usage, 0.94 for AF burden, and 0.72 for AF severity. Test-retest reliability at three months stands at r = 0.71, r = 0.75, and r = 0.64 for health care usage, AF burden, and AF severity respectively [28].

### Data collection

Patients with PAF provided details on their age, sex, educational level, and employment status. The data collection was completed at a visit with administrative staff. Additionally, we extracted clinical data from medical records.

### Data analysis

We employed IBM SPSS Statistics, version 26, for all analyses (IBM, Armonk, NY, USA). For baseline continuous variables withed a normal distribution, we used T tests to compare the CBT and TAU groups. We analyzed categorical data using the chi-squared test or Fisher’s exact test. For data that did not conform to a normal distribution, we described it using the median and interquartile range (IQR) and employed Mann–Whitney’s U test to assess differences between the CBT and TAU groups. We evaluated the efficacy of CBT by examining the HRQoL score differences between the groups across three time points, taking into account baseline covariates, using Generalized Estimating Equations (GEE).

## Results

### Drop out

At the end of the 10th week, 38 patients in CBT group had completed the trial, and 7 patients had dropped out. The reasons were as follows: two dropped out due to changing their usual residence, two discontinued treatment because of unsatisfactory results, two dropped out because of prepare for catheter ablation, and one person withdrew because of a fall that required hospital treatment. In the TUA, 42 people completed the treatment and 3 people dropped out. Of them, two people voluntarily withdrew from the study, and one patient was lost of follow-up. At the end of the 6-month follow up, in CBT group 35 patients had completed the follow up. Two patients were lost to follow-up because they did not answer the phone, and one declined the follow-up invitation. In TUA group, 37 patients had completed the follow up, five patients lost to follow-up because they were not contacted.

### Demographic and clinical characteristics

In CBT group, the patients’ mean age was 60.78 years (SD = 7.5), and 30 patients (66.7%) were male. In TAU group, the mean age was 61.67 years (SD = 6.0), and 22 patients (66.7%) were male There was no significant difference between the CBT and TUA groups in terms of sociodemographics and clinical characteristics (*P* > 0.05). (Table 2).

**Table 2:** Patient Characteristics

	CBT (N = 45)	TAU(N = 45)	t/χ^2^/Z	p
Age, mean years, mean (IQR)	60.78(7.50)	61.67(5.78)	-0.259	0.796
Gender, n (% male)	30(66.7%)	22(48.9%)	2.915	0.088
Work status			0.216	0.642
Employed	14(31.1%)	12(26.7%)		
Unemployed	31(68.9%)	33(73.3%)		
marital status			0.278	0.598
single	8(17.6%)	9(22.2%)		
married	37(82.4%)	35(77.8%)		
Education			0.119	0.942
Elementary school	8(17.8%)	7(15.6%)		
High school	9(20%)	10(22.2%)		
College/university	28(62.2%)	28(62.2%)		
Monthly income(¥)			0.278	0.870
<5000	16(35.6%)	17(37.8%)		
5000 ~ 10,000	19(42.2%)	20(44.4%)		
>10,000	10(22.2%)	8(17.8%)		
Duration of illness			0.080	0.777
< 1 years	37	38		
≥ 1 years	8	7		
Comorbidities			1.766	0.622
Arterial hypertension	10	8		
Diabetes mellitus	4	4		
Ischaemic heart disease	9	10		
Heart failure	0	0		
Obstructive sleep apnea	4	1		
Previous stroke/TIAd(transient ischemic attack)	0	1		
Current medication, n (%)			5.210	0.074
Antiarrhythmics	3	11		
Beta blockers	32	32		
Anticoagulation	20	15		
SF-12MCS, mean (SD)	37.317(7.15)	34.268(7.17)	2.019	0.047
SF-12PCS, median (IQR)	39.3(9.15)	39(13.5)	-0.303	0.762
GAD-7, median (IQR)	6 (6)	10(8)	-1.084	0.071
PHQ-9, median (IQR)	10(9)	12(11.5)	-0.598	0.55
BIPQ, median IQR)	46(8)	48(9)	-1.457	0.145
AFSS symptoms scale, median (IQR)	11(3.5)	11(3)	-0.542	0.588

At baseline, no significant difference was observed between the CBT and TAU group in terms of the SF-12 PCS (Z=-0.303, *p* = 0.762). However, the CBT group exhibited a higher SF-12 MCS score compared to the TAU group (37.317 ± 7.151 vs. 34.268 ± 7.170, *p* = 0.047). After adjusting for baseline covariates such as age, gender, education, monthly income, employment status and the initial SF-12 MCS scores, the generalized estimating equation indicated a significant increase over time in both SF-12 PCS (Wald χ^2^ (2) = 27.507, *p* < 0.001) and SF-12 MCS (Wald χ2 (2) = 191.373, *p* < 0.001). Significant group effect were evident in SF-12 MCS (β = 5.053, 95%CI (2.766, 7.34), *p* < 0.001) and SF-12 PCS (β=-3.277,95%CI (-5.745, -0.809), *p* = 0.009). Apart from the PHQ-9 score, between-group effects were observed in GAD-7, BIPQ and AFSS symptoms scales. For a detailed breakdown, refer to (Table 3).

**Table 3:** Generalized estimating equation predicting all assessments by time and group

	Between group					Within group				
	B	SE	95% CI		*p*	B	SE	95% CI		*p*
			Lower	Upper				Lower	Upper	
SF-12 PCS	-3.277	1.260	-5.745	-0.809	0.009	-6.047	1.487	-8.960	-3.133	<0.001
SF-12 MCS	5.053	1.167	2.766	7.340	<0.001	-10.94	1.3141	-13.515	-8.364	<0.001
GAD-7	-3.872	0.547	-4.943	-2.800	<0.001	0.889	0.467	-0.026	1.804	0.057
PHQ-9	-1.990	1.028	-4.004	0.025	0.053	1.480	0.391	0.713	2.247	<0.001
BIPQ	-3.356	0.537	-4.408	-2.304	<0.001	11.374	1.157	9.106	13.643	<0.001
AFSS symptoms scale	-1.040	0.308	-1.644	-0.437	0.001	-0.039	0.345	-0.715	0.636	0.909

### Primary and secondary outcome

At the 6-month follow-up, the CBT group exhibited a higher SF-12 Mental Component Score (MCS) of 50.261 ± 0.758 compared to 45.208 ± 0.887 in the TAU group, *p* < 0.001. Additionally, the CBT group had lower scores for GAD-7 (4.150 ± 0.347 vs. 8.022 ± 0.423, *p* < 0.001), BIPQ (34.700 ± 0.432 vs. 38.026 ± 0.318, *p* < 0.001), and AFSS (9.890 ± 0.217 vs. 10.928 ± 0.218, *p* = 0.001). In contrast, the SF-12 PCS scores (44.212 ± 0.816 vs. 47.489 ± 0.960, *p* = 0.139) and the PHQ-9 scores (8.419 ± 0.713 vs. 10.409 ± 0.741, *p* = 0.794) scores were comparable between both groups. Refer to (Table 4) for a detailed breakdown.

**Table 4:** All assessments scores overtime and between-group differences at 6-month follow-up

	Baseline (M ± SE)		Post-treatment (M ± SE)		6-month Follow-up (M ± SE)		Between-group differences at 6month follow-up	
	CBT	TAU	CBT	TAU	CBT	TAU	Estimated mean change (95%CI)	p
SF-12 PCS	40.216±0.935	41.442±1.217	42.113±0.875	42.969±1.189	44.212±0.816	47.489±0.960	-3.277 (-6.974, 0.42)	0.139
SF-12 MCS	37.317±1.054	34.268±1.058	48.579±0.763	40.036±0.894	50.261±0.758	45.208±0.887	5.053 (1.628, 8.478)	<0.001
GAD	7.311±0.570	8.911±0.616	4.614±0.428	8.636±0.535	4.150±0.347	8.022±0.423	-3.872 (-5.476, -2.270)	<0.001
PHQ	11.111±0.916	11.889±0.885	9.568±0.822	11.004±0.697	8.419±0.713	10.409±0.741	-1.990 (-5.010,1.027)	0.794
BIPQ	45.133±0.941	49.40±1.196	38.679±0.603	46.532 ± 0.754	34.700±0.432	38.026±0.318	-3.356 (-4.931, -1.781)	<0.001
AFSS symptoms scale	11.222±0.370	10.890±0.321	10.525±0.228	11.280±0.217	9.890±0.217	10.928±0.218	-1.040 (-1.944, -0.137)	0.011

The GEE analysis for each item of the BIPQ score found significant between-group differences in items of consequences (*p* < 0.001), treatment control (*p* < 0.001), illness concern (*p* = 0.019) and understanding (*p* < 0.001). In addition, all items had a significant time effect. These data are presented in detail in Table 5.

**Table 5:** Generalized estimating equation analysis of the BIPQ items scores by time and group

	Between group			Within group		
	B	Wald χ^2^	*p*	B	Wald χ^2^	*p*
Consequences	-0.926	17.636	<0.001	1.36	78.861	<0.001
Timeline	0.376	0.519	0.471	1.298	33.992	<0.001
Personal control	-0.559	0.293	0.588	-1.155	11.774	0.003
Treatment control	0.851	17.912	<0.001	-0.847	7.941	0.019
Identity	-0.274	0.249	0.618	0.507	17.125	<0.001
Illness concern	-0.592	5.535	0.019	2.446	112.067	<0.001
Understanding	1.486	30.162	<0.001	-1.983	200.863	<0.001
Emotional response	-0.144	21.391	0.502	1.726	39.101	<0.001

## Discussion

Our findings demonstrate that while both interventions led to an overall improvement in patients’ mental component of Health-Related Quality of Life (HRQoL) over time, Cognitive Behavioral Therapy (CBT) elicited a more significant improvement post-treatment. This aligns with prior research indicating that mindfulness-based CBT can enhance patients’ quality of life up to six months post-intervention [29]. While several previous studies have shown quality of life improvements across both physical and mental components in refractory patients undergoing radiofrequency ablation [30, 31], our study did not highlight a therapeutic advantage for physical components of HRQoL. This discrepancy could be attributed to our study participants’ stable conditions, where fundamental treatment was sufficient to aid the recovery of physical function. A study by Pathak et al. reported that the HRQoL of patients can be improved comprehensively with the targeted management of various AF risk factors. Our results, consistent with that study, suggest that comprehensive interventions, involving physical and mental dimensions, improve the HRQoL of patients with AF [32]. The “hard” clinical endpoints such as stroke and death should not be the sole indicators of treatment success; patient perspective must also be emphasized.

Incorporating psychotherapy into the comprehensive management of atrial fibrillation (AF) patients may contribute to reducing the disease treatment burden [16, 33, 34].At the endpoint, the CBT group exhibited significant improvements in anxiety symptoms, measured using the GAD-7, a change not mirrored in the control group. This corroborates findings from previous studies on Cardiovascular Disease (CVD) where CBT led to an immediate reduction in anxiety compared to control conditions [35, 36]. As supported by robust evidence, CBT is effective in alleviating anxiety symptoms [37, 38]. In this study, the CBT module was tailored to the characteristics of AF and proved to be efficacious in reducing anxiety in AF patients. Interestingly, we also found an improvement in the assessment of AF symptoms as measured by the AF Symptom Severity (AFSS) after the CBT intervention exclusively. This change could be interpreted from both symptom and underlying pathophysiological mechanism perspectives. Given that AF symptoms, which include both heart-specific symptoms and nonspecific symptoms, overlap with anxiety symptoms, CBT could indirectly improve AF symptoms by reducing anxiety. Additionally, anxiety disorders often manifest hyperactivity of the sympathetic nervous system and the hypothalamic-pituitary-adrenal (HPA) axis [39, 40], potentially increasing cardiotoxicity and inducing systemic inflammatory responses implicated in arrhythmias [41–43]. Therefore, anxiety is deemed an independent risk factor for AF, and future research should examine whether anxiety mediates the improvement of AF symptoms following CBT.

Contradicting our findings, previous research revealed no difference between CBT and Treatment as Usual (TAU) groups concerning changes in depressive symptoms [44]. This discrepancy may stem from our treatment protocol focusing more on improving anxiety symptoms than depressive symptoms. It’s important to note that long-term psychological interventions are typically necessary to address depressive symptoms effectively. Reavell et al. found that CBT can improve depressive symptoms in CVD [45], which is inconsistent with our results. The inconsistency in conclusions may be due to the different study protocols, as our treatment focused more on improving anxiety symptoms.

Earlier studies have reported that CBT improves illness perceptions in patients with non-cardiac chest pain and benign palpitations in the short and long term [46]. Likewise, improvement in symptom preoccupation in AF patients following exposure-based therapy persisted through a 6-month follow-up period. Such findings are consistent with our own. We also found more significant improvements in the CBT than in the TAU group in terms of disease consequences, treatment control, disease concern, and understanding of disease, confirming our hypothesis that CBT can correct patients’ distorted disease perception and increase their self-efficacy, enabling patients to focus less on symptoms and reduce avoidance behaviors. Furthermore, we suggest that GAD might be a more appropriate reference than item emotion response of the BIPQ for judging patients’ mood changes, despite the similar findings between our groups regarding the emotional response. Illness perception is also associated with medication adherence [13] and psychological distress [47]. Changes in illness perception may also be linked to increased interpersonal support and skills gained from CBT that are lacking in TAU [48], but further investigation is needed to substantiate this theory.In terms of study feasibility and acceptability, our attrition rate was relatively low at 15.6%, slightly higher than rates reported in similar studies. Participant feedback suggested that travel distance for in-person sessions was a hindrance.

In addition, we make a preliminary estimate of the time costs. The initial evaluation lasted 0.5 h, the therapist preparation time for each session was 0.5 h, the time of each treatment session 1 h, the supervision every 5 weeks approximately 2.5 h, and the telephone notification 10-15 min per person. Therefore, the total time each therapist needed to devote to individual CBT was approximately18.75 h for every course of treatment. However, the long-term investment was worthwhile, as the results the patients had a more significant improvement in quality of life after CBT invention than without it, and the effect was maintained 6 months later.

Limitations of our study include a single-center study design, and potential subject bias. The lack of randomization and a younger sample than the general AF population restrict the generalizability of our findings. Additionally, the absence of continuous heart rhythm measurements using an objective heart rate-checking device and an assessment of the objective AF burden for accurately determining the occurrence of AF symptoms represents another limitation.

## Conclusion

The findings of our study underscore the significant role of cognitive-behavioral therapy (CBT) in enhancing the mental component of Health-Related Quality of Life (HRQoL) for patients with atrial fibrillation (AF). Our study showed that despite various interventions, it is the inclusion of CBT that potentially leads to notable improvements in patient outcomes. Importantly, our study revealed that CBT could significantly alleviate anxiety symptoms and also positively impacted the subject assessment of AF symptoms in AF patients. These results offer valuable insights into the correlation between anxiety symptoms and AF-specific manifestations, highlighting the potential of CBT in managing these intertwined conditions. Additionally, our findings suggested a possible shift in patient’s illness perceptions following a CBT intervention. In conclusion, while acknowledging the limitations, our study indicates the significant potential of integrating CBT in the therapeutic regimen for patients with AF. Such an integration could lead to substantial improvements in HRQoL, reduce anxiety, and improve symptom management, thus setting a promising direction for future research in this area.

## Data Availability

Please contact author for data requests.

## Abbreviations

AF: Atrial fibrillation
PAF: Paroxysmal atrial fibrillation
HRQoL: Health-Related Quality of Life
CBT: Cognitive Behavioural Therapy
SF-12: Item Short Form Health Survey
MCS: Mental Component Score
PCS: Physical Component Score
GAD-7: General Anxiety Disorder-7
PHQ-9: Patient Health Questionnaire-9
AFSS: University of Toronto Atrial Fibrillation Severity Scale
BIPQ: Brief Illness Perception Questionnaire
CVDs: Cardiovascular Diseases
GEE: Generalized Estimating Equations
